# Supplementary material for: Prevalence and molecular heterogeneity of glucose-6-phosphate dehydrogenase (G6PD) deficiency in the Senoi Malaysian Orang Asli population
Source: PLoS One. 2023 Dec 12;18(12):e0294891. doi: 10.1371/journal.pone.0294891 (PMC10715666; doi:10.1371/journal.pone.0294891)
Supplement: S1 File — The study’s minimal underlying data set. (PDF) [file pone.0294891.s001.pdf]

SAMPLE ID	GENDER	ETHNIC	AGE	G6PD (U/g	%AMM	Hb	RBC	MCH	MCV
SCM001	Male	Che Wong	62	9.1	77%	13.2	4.53	29.1	92.9
SCM002	Male	Che Wong	21	9.4	80%	15	5.29	28.4	89.9
SCM003	Male	Che Wong	19	8.1	69%	13.6	4.41	30.9	96.4
SCF005	Female	Che Wong	32	11.0	93%	11	4.84	22.8	76.1
SCF006	Female	Che Wong	23	11.7	99%	10	3.43	29.2	92.1
SCF007	Female	Che Wong	17	9.8	83%	12	4.32	27.7	88.5
SCF008	Female	Che Wong	39	11.3	96%	13.9	4.88	28.5	88.4
SJM001	Male	Jahut	43	18.4	156%	7.1	3.49	20.3	70
SJM002	Male	Jahut	25	1.0	9%	16.5	5	33	98.9
SJM003	Male	Jahut	43	1.0	8%	13.5	4.75	28.4	89.1
SJM005	Male	Jahut	19	11.8	100%	14.6	4.84	30.1	90.1
SJM006	Male	Jahut	18	11.1	94%	14.6	5.44	26.8	82.2
SJM008	Male	Jahut	42	9.7	82%	15.4	4.51	34.3	103.3
SJM010	Male	Jahut	35	2.0	17%	14.6	4.05	36	110.6
SJF081	Female	Jahut	76	8.8	74%	10.6	3.1	34.1	108.9
SJF082	Female	Jahut	48	2.0	17%	13.6	4.2	32.3	99.1
SJF083	Female	Jahut	41	10.7	90%	11	3.84	28.7	89.6
SJM012	Male	Jahut	22	10.8	92%	14.9	5.13	29.1	91.5
SJM013	Male	Jahut	16	3.0	25%	13.6	4.45	30.6	93.7
SJF085	Female	Jahut	56	1.4	12%	13.5	4.41	30.6	95.4
SJF086	Female	Jahut	46	0.9	8%	12.2	4.37	28	89.4
SJM015	Male	Jahut	58	11.9	101%	12.9	4.14	31.2	99.2
SJF087	Female	Jahut	26	1.0	8%	11.1	3.85	29	92.3
SJM016	Male	Jahut	25	12.8	108%	16.2	6.04	26.8	83
SJF089	Female	Jahut	57	12.5	106%	11.6	4.78	24.2	76.5
SJF090	Female	Jahut	48	8.4	71%	6.1	3.77	16.1	59.5
SJM018	Male	Jahut	32	8.7	73%	14.4	4.79	30.1	95.7
SJF094	Female	Jahut	37	11.7	99%	12.6	4.81	26.2	78.6
SJF095	Female	Jahut	47	10.8	92%	12.5	4.43	28.1	88.5
SJM019	Male	Jahut	52	10.1	86%	15.3	4.89	31.2	94.3
SJM022	Male	Jahut	8	13.1	111%	11.7	3.88	30.3	91.8
SJF100	Female	Jahut	30	12.2	104%	13	4.87	26.7	83.3
SJF101	Female	Jahut	12	13.7	116%	11.2	4.46	25.2	79.5
SJM025	Male	Jahut	52	11.0	94%	15.2	5.86	26	80.7
SJF104	Female	Jahut	49	12.9	110%	12.9	6.39	20.2	68.8
SJM027	Male	Jahut	2	14.0	119%	9.1	4.67	19.5	63.8
SJF105	Female	Jahut	29	19.5	165%	9.2	5.34	17.2	60.3
SJM028	Male	Jahut	25	10.2	86%	14.7	5.91	24.8	78.3
SJM030	Male	Jahut	39	11.1	94%	15.3	5.53	27.7	90.7
SJF110	Female	Jahut	37	0.7	6%	10.5	4.02	26.2	85.8
SJF111	Female	Jahut	28	17.8	151%	9.4	4.74	19.9	71
SJF114	Female	Jahut	49	9.1	77%	12	3.78	31.6	102
SJF115	Female	Jahut	56	10.4	88%	13.1	4.43	29.6	92.6
SJM031	Male	Jahut	76	11.9	101%	13	4.34	30.1	96.4
SJM032	Male	Jahut	19	10.5	89%	14.5	4.85	30	92
SJF117	Female	Jahut	57	10.2	87%	14.1	4.49	31.4	94.9

SJM034	Male	Jahut	25	0.7	6%	14.4	5.04	28.5	92.3
SJM035	Male	Jahut	17	10.2	86%	16.1	5.08	31.8	96.3
SJM036	Male	Jahut	23	1.0	8%	11.9	3.99	29.8	88.4
SJM037	Male	Jahut	47	10.6	90%	14.8	4.48	33	101.9
SJM038	Male	Jahut	36	10.3	87%	16.2	4.86	33.2	100.1
SJF124	Female	Jahut	66	6.3	53%	10.8	3.29	32.7	106.6
SJF126	Female	Jahut	44	11.0	93%	13.1	4.19	31.3	97.9
SJM040	Male	Jahut	45	0.8	7%	14.3	5.16	27.6	87.6
SJM045	Male	Jahut	11	13.6	115%	11.4	5.26	21.6	69
SJM049	Male	Jahut	37	13.4	114%	14.6	5.44	26.8	84.3
SJF137	Female	Jahut	28	12.4	105%	12.8	3.51	26.5	102.4
SJM058	Male	Jahut	13	15.4	130%	11.2	4.28	26.3	81.1
SJF142	Female	Jahut	11	8.8	75%	12.5	4.65	26.9	82.2
SJM065	Male	Jahut	34	11.6	98%	13.4	4.84	27.6	86.5
SJM066	Male	Jahut	25	13.5	114%	12.8	5.43	23.5	79.2
SJM067	Male	Jahut	42	10.7	90%	15.2	4.1	37.2	114.2
SJM068	Male	Jahut	52	10.8	92%	14.6	5.95	24.4	82.3
SJM072	Male	Jahut	11	11.6	98%	12.2	4.84	25.3	78.1
SJM077	Male	Jahut	16	2.5	21%	12.9	5.34	24.1	81.8
SJF157	Female	Jahut	43	9.8	83%	10.5	4.1	25.6	81.2
SJF160	Female	Jahut	6	16.7	142%	10.2	5.24	19.5	63.6
SJM163	Male	Jahut	33	2.9	24%	13.9	4.43	31.5	93.5
SMM001	Male	Mah Meri	60	3.2	27%	16.5	5.49	30	92.8
SMM002	Male	Mah Meri	21	2.9	24%	15.9	4.82	33.1	101.5
SMM003	Male	Mah Meri	17	2.8	24%	15.9	5.69	27.9	88.2
SMM004	Male	Mah Meri	26	13.6	115%	14.8	5.53	26.8	84.6
SMF013	Female	Mah Meri	61	15.6	132%	13.6	4.64	29.2	90.9
SMM005	Male	Mah Meri	27	12.8	109%	13.7	5.13	26.7	88.3
SMM006	Male	Mah Meri	60	12.4	105%	14.1	4.39	32.1	100.4
SMM007	Male	Mah Meri	20	12.3	105%	15.4	6.15	25	80.3
SMF014	Female	Mah Meri	29	20.1	170%	8.6	4.05	21.2	71.2
SMF012	Female	Mah Meri	37	6.4	54%	13.5	5.02	26.8	82.9
SMF016	Female	Mah Meri	44	12.8	108%	13.4	4.8	27.9	83.6
SMF017	Female	Mah Meri	57	12.7	108%	13.1	4.41	29.6	90.5
SMF038	Female	Mah Meri	40	11.5	97%	12.7	4.47	28.4	87.6
SMF039	Female	Mah Meri	32	14.5	123%	12.1	4.87	24.8	76.3
SMM011	Male	Mah Meri	48	10.8	92%	14.8	5.37	27.5	85.8
SMF019	Female	Mah Meri	28	17.5	149%	10	4.6	21.8	72
SMF022	Female	Mah Meri	27	11.3	95%	12.9	4.86	26.5	82.8
SMF023	Female	Mah Meri	24	17.1	145%	12.4	5.19	23.9	78.1
SMF024	Female	Mah Meri	40	13.4	114%	14.1	4.77	29.6	91.3
SMF025	Female	Mah Meri	26	14.6	124%	13.8	5.18	26.7	82.1
SMF026	Female	Mah Meri	42	13.9	118%	13.4	4.72	28.3	86.1
SMF027	Female	Mah Meri	20	13.8	117%	11.3	4.26	26.5	84.9
SMF028	Female	Mah Meri	50	23.3	197%	6.8	3.73	18.3	63.9
SMF029	Female	Mah Meri	21	14.9	126%	11.6	4.82	24.1	80.2
SMF030	Female	Mah Meri	25	12.3	105%	13.6	4.3	31.5	93.8

SMF032	Female	Mah Meri	26	14.4	122%	13.7	4.79	28.6	88.3
SMF034	Female	Mah Meri	61	15.2	128%	12.1	3.88	31.3	99.8
SMF037	Female	Mah Meri	18	15.7	133%	13.4	4.65	28.9	88.9
SMF040	Female	Mah Meri	31	13.8	117%	13.9	4.25	32.6	100.3
SMF041	Female	Mah Meri	79	20.6	174%	9.1	4.63	19.7	67.8
SMF042	Female	Mah Meri	40	12.3	104%	13.2	4.86	27.3	85.9
SMF043	Female	Mah Meri	34	18.0	153%	9.5	4.9	19.4	65.1
SMF044	Female	Mah Meri	24	13.2	112%	12.1	5.69	21.2	70.7
SMF046	Female	Mah Meri	36	21.3	180%	7.3	4.56	16	58.5
SSF075	Female	Semai	29	3.7	31%	11.8	4.46	26.5	82.2
SSF077	Female	Semai	30	19.1	162%	10.3	4.64	22.3	72.4
SSM002	Male	Semai	38	10.0	84%	14.8	4.86	30.4	88.4
SSM004	Male	Semai	50	11.9	101%	14.5	5.88	24.7	74.4
SSF080	Female	Semai	27	5.9	50%	12.9	4.84	26.7	85.2
SSM005	Male	Semai	50	8.8	74%	16	5.46	29.3	92.3
SSM006	Male	Semai	50	11.3	96%	13.3	4.44	30	87.3
SSM007	Male	Semai	34	2.1	18%	13.7	4.73	29	85.4
SSM008	Male	Semai	53	14.5	123%	13	5.65	23	70
SSM009	Male	Semai	41	2.4	20%	14	5	28	82.3
SSF083	Female	Semai	38	13.0	110%	13.4	5.14	26.2	83.5
SSF084	Female	Semai	36	10.1	86%	13.7	4.59	29.8	92.4
SSF085	Female	Semai	22	8.5	72%	13.2	4.4	30.1	93.3
SSF086	Female	Semai	25	3.8	32%	11.9	4.09	29.2	91
SSF087	Female	Semai	30	11.2	95%	12.9	4.99	25.9	81.4
SSM010	Male	Semai	52	12.8	109%	14.5	5.64	25.7	79.9
SSF088	Female	Semai	29	11.6	98%	14	5.67	24.6	76.7
SSM011	Male	Semai	29	3.3	28%	15	5.55	27	85.4
SSF089	Female	Semai	42	11.2	95%	13	4.63	28.1	84.9
SSF090	Female	Semai	37	9.3	79%	12.7	4.95	25.6	80.5
SSF091	Female	Semai	28	10.7	91%	13.3	4.62	28.7	90.6
SSF092	Female	Semai	26	11.9	101%	13.8	5.05	27.3	85.6
SSF093	Female	Semai	41	10.0	85%	10.6	5.25	20.2	64.9
SSF094	Female	Semai	43	9.1	77%	11.9	4.43	26.8	84.8
SSM012	Male	Semai	40	10.0	85%	15.4	5.06	30.4	94.4
SSF095	Female	Semai	42	7.7	65%	13.3	4.32	30.8	97.1
SSF096	Female	Semai	33	5.9	50%	11.9	3.63	32.7	99.9
SSM013	Male	Semai	42	0.8	7%	14.2	5.09	27.9	88
SSM014	Male	Semai	52	10.6	90%	14.1	4.32	32.7	95.9
SSM015	Male	Semai	43	10.3	87%	15.4	5.16	29.9	87.5
SSF097	Female	Semai	32	11.9	101%	14.1	5.45	25.8	78.8
SSF098	Female	Semai	40	11.9	100%	13.3	4.77	27.9	82.6
SSM016	Male	Semai	46	11.5	97%	14.5	4.46	32.6	93.4
SSF099	Female	Semai	35	17.0	144%	10	4.64	21.5	71.2
SSF100	Female	Semai	33	9.4	80%	12	4.17	28.9	88.2
SSF101	Female	Semai	41	8.9	76%	12.5	5.09	24.6	80.3
SSF102	Female	Semai	36	10.4	88%	13.1	4.5	29	89.6
SSM017	Male	Semai	47	14.8	125%	13.8	5.56	24.8	79.3

SSF104	Female	Semai	35	3.5	29%	13.7	5.06	27.1	87.1
SSF105	Female	Semai	32	10.5	89%	12.1	4.2	28.7	87.6
SSM018	Male	Semai	53	1.1	9%	12.8	4.6	27.7	82
SSM019	Male	Semai	51	16.5	140%	10.3	5.14	20	68.5
SSF106	Female	Semai	29	13.9	118%	13.1	4.37	29.9	94.3
SSF107	Female	Semai	34	13.9	118%	12.5	4.57	27.4	85.8
SSM020	Male	Semai	37	0.8	7%	13.1	4.12	31.7	98
SSF108	Female	Semai	41	10.5	89%	12.5	3.88	32.3	97.1
SSF110	Female	Semai	25	11.8	100%	12.1	4	30.2	94.2
SSF111	Female	Semai	30	13.5	114%	13.9	5.31	26.1	81.5
SSM021	Male	Semai	55	11.7	99%	14.3	4.89	29.3	90.9
SSM022	Male	Semai	48	2.7	23%	12.4	4.81	25.7	76.7
SSM023	Male	Semai	71	17.6	149%	8.3	4.46	18.6	66
SSM024	Male	Semai	38	11.1	94%	14.6	5.18	28.1	89.3
SSF112	Female	Semai	44	12.6	107%	11.6	4.33	26.7	79.4
SSF113	Female	Semai	47	12.0	101%	14.5	4.94	29.3	86.1
SSF115	Female	Semai	53	9.6	81%	15.4	4.72	32.6	94.2
SSM025	Male	Semai	13	13.1	111%	14.7	5.36	27.5	86.3
SSF114	Female	Semai	44	10.5	89%	14.3	4.57	31.3	98.9
SSF116	Female	Semai	36	13.4	114%	13.1	4.43	29.5	90.6
SSF117	Female	Semai	38	12.0	101%	12.6	4.68	26.9	80.3
SSF118	Female	Semai	32	11.6	98%	13.2	4.47	29.4	86.5
SSM026	Male	Semai	50	13.8	117%	14.7	4.88	30.1	89.4
SSM027	Male	Semai	38	2.6	22%	14.4	4.51	31.9	95.6
SSF119	Female	Semai	33	12.4	105%	12.6	4.3	29.3	86.5
SSF120	Female	Semai	36	11.1	94%	14	4.69	29.9	85.3
SSF122	Female	Semai	14	10.2	87%	11.9	4.42	26.9	86.4
SSF124	Female	Semai	31	10.3	87%	12.6	5.2	24.3	79.8
SSM029	Male	Semai	58	12.3	104%	13.8	4.94	28	82.9
SSF133	Female	Semai	24	12.1	102%	12.8	5.09	25.1	79.9
SSM030	Male	Semai	50	13.5	114%	15.1	5.89	25.7	77.2
SSF134	Female	Semai	52	12.9	109%	14.6	5.41	27	80.7
SSM031	Male	Semai	42	2.6	22%	14.7	4.42	33.3	96.4
SSF135	Female	Semai	43	1.0	8%	12.2	4.73	25.8	75.7
SSF136	Female	Semai	29	12.2	103%	12.9	4.94	26	83.1
SSM032	Male	Semai	33	10.2	86%	14.8	4.85	30.4	94.9
SSF137	Female	Semai	27	2.6	22%	14.1	4.42	32	97.7
SSF138	Female	Semai	51	10.3	88%	13.3	4.23	31.4	93.1
SSF139	Female	Semai	38	14.8	125%	14.2	4.61	30.8	93.5
SSF140	Female	Semai	28	9.6	81%	14.7	4.87	30.2	92.8
SSM033	Male	Semai	28	10.5	89%	16.3	5.58	29.2	91.8
SSF141	Female	Semai	34	9.0	77%	12.9	5.21	24.8	80.1
SSM034	Male	Semai	41	12.0	102%	15.2	5.46	27.9	90.1
SSF145	Female	Semai	24	15.5	132%	12.3	5.1	24.2	77.8
SSF147	Female	Semai	50	1.1	9%	10.6	4.24	24.9	81.8
SSM038	Male	Semai	24	2.6	22%	13.6	5.67	24	79.3
SSM039	Male	Semai	35	13.8	117%	14.6	5.81	25.2	78.5

SSF149	Female	Semai	45	12.2	103%	13.4	4.52	29.5	90.8
SSF150	Female	Semai	39	5.9	50%	11.9	3.91	30.4	93.6
SSM040	Male	Semai	56	12.3	104%	14.1	4.24	33.2	95.9
SSM041	Male	Semai	48	11.9	101%	15.4	5.36	28.7	86.1
SSM042	Male	Semai	33	12.0	102%	14.4	4.61	31.4	97.2
SSM043	Male	Semai	31	10.9	92%	13.9	4.98	27.8	86.3
SSM044	Male	Semai	43	12.0	102%	14	4.6	30.4	92.7
SSF151	Female	Semai	64	12.1	103%	14.2	4.61	30.8	95.7
SSF153	Female	Semai	20	14.9	126%	11.4	4.41	25.9	84
SSF154	Female	Semai	3	12.4	105%	12.1	4.18	29.1	89.1
SSM047	Male	Semai	44	12.7	108%	15.8	5.02	31.5	96.9
SSF156	Female	Semai	18	15.1	128%	9.2	4.06	22.7	75
SSM049	Male	Semai	28	11.8	100%	14.9	4.83	30.8	94.7
SSM050	Male	Semai	53	11.3	96%	14.3	5.17	27.7	87.7
SSF157	Female	Semai	55	8.7	74%	13.9	4.53	30.7	94
SSF159	Female	Semai	34	8.2	70%	11.3	4.44	25.4	79.9
SSF160	Female	Semai	74	10.4	89%	14	5.17	27	86.6
SSM054	Male	Semai	22	13.9	118%	15.8	6.1	25.9	84
SSF161	Female	Semai	53	12.1	103%	14.5	5.68	25.5	81.8
SSF164	Female	Semai	23	13.3	113%	13.3	4.59	29.1	91.4
SSM055	Male	Semai	46	13.0	111%	14	5.79	24.3	78
SSM056	Male	Semai	38	2.8	24%	13.3	4.1	32.3	102.1
SSF166	Female	Semai	45	8.6	73%	12.4	4.16	29.7	96.3
SSM059	Male	Semai	23	10.1	86%	16.3	5.42	30	96
SSM060	Male	Semai	28	10.0	85%	15.5	4.95	31.3	97.1
SSM062	Male	Semai	37	12.6	107%	13.7	4.46	30.7	95.1
SSM063	Male	Semai	50	11.6	98%	14.4	4.91	29.4	92.5
SSM064	Male	Semai	15	12.5	106%	13.5	5.01	26.9	86
SSF168	Female	Semai	36	10.1	86%	12.7	4.26	29.7	94.4
SSF171	Female	Semai	11	10.4	88%	12.1	4.37	27.7	87.4
SSM068	Male	Semai	19	14.0	119%	12.9	5.21	24.8	81.3
SSM069	Male	Semai	9	20.2	171%	8.1	4.37	18.5	65.6
SSF173	Female	Semai	16	3.8	32%	13.4	5.08	26.3	83.6
SSF172	Female	Semai	40	10.6	90%	13.4	4.23	31.6	93.2
SSF174	Female	Semai	43	11.8	100%	14.3	4.79	29.9	92.1
SSF176	Female	Semai	44	9.4	79%	13.7	4.61	29.7	91.7
SSF177	Female	Semai	21	8.9	76%	12.2	4.21	29	92.3
SSF178	Female	Semai	39	11.8	100%	14	4.57	30.6	93.9
SSF179	Female	Semai	25	12.4	105%	12.6	4.93	25.5	82.4
SSM071	Male	Semai	30	12.3	104%	15.8	5.14	30.7	95.8
SSF180	Female	Semai	63	12.5	106%	13.1	4.37	30.1	91.1
SSF182	Female	Semai	33	11.8	100%	12.4	4.16	29.9	94
SSM073	Male	Semai	65	14.1	119%	11.3	4.81	23.4	75.9
SSF185	Female	Semai	67	18.3	155%	7	3.52	19.9	69.8
SSF186	Female	Semai	34	9.1	77%	11.6	4.59	25.3	79.4
SBM001	Male	Semoq Bei	50	0.8	7%	6.8	2.52	27	69.6
SBM003	Male	Semoq Bei	25	9.6	81%	14.6	5.05	29	92.7

SBM004	Male	Semoq Bei	27	10.0	85%	14.1	4.6	30.7	95.4
SBF032	Female	Semoq Bei	38	10.1	86%	13.7	4.51	30.3	90.1
SBM005	Male	Semoq Bei	52	9.0	76%	12.3	4.05	30.3	94.7
SBF033	Female	Semoq Bei	60	8.3	71%	13.7	4.79	28.5	91
SBM006	Male	Semoq Bei	36	8.2	69%	15.8	5.4	29.3	94.2
SBM007	Male	Semoq Bei	20	10.1	86%	17.1	5.32	32.1	99
SBF034	Female	Semoq Bei	53	10.0	85%	14.2	4.85	29.3	91.8
SBM010	Male	Semoq Bei	58	9.2	78%	15.3	5.49	27.8	88.9
SBM011	Male	Semoq Bei	30	13.4	114%	15.1	6.73	22.4	73.6
SBM014	Male	Semoq Bei	28	9.7	82%	14.6	5.02	29	88.5
SBF037	Female	Semoq Bei	32	9.7	82%	13.6	4.66	29.1	90.5
SBM015	Male	Semoq Bei	24	10.9	93%	17.9	5.76	31.1	97.6
SBM017	Male	Semoq Bei	50	9.7	82%	14.3	4.72	30.3	94
SBM018	Male	Semoq Bei	22	8.4	71%	16.2	5.39	30	95.4
SBM019	Male	Semoq Bei	24	9.9	84%	13.1	5.23	25	78.4
SBF038	Female	Semoq Bei	52	9.4	79%	11.4	4.2	27.2	87.7
SBF039	Female	Semoq Bei	30	8.1	69%	12.9	4.15	31	94.8
SBM020	Male	Semoq Bei	35	8.6	73%	16.1	5.13	31.3	95.6
SBM021	Male	Semoq Bei	18	12.9	109%	12.4	4.86	25.4	80.5
SBF040	Female	Semoq Bei	15	8.0	68%	12.9	4.61	28	90.3
SBF041	Female	Semoq Bei	22	9.9	84%	10.5	3.43	30.6	97.4
SBM022	Male	Semoq Bei	16	10.5	89%	13.3	4.93	26.9	85.1
SBF042	Female	Semoq Bei	38	12.6	107%	11.9	4.29	27.8	90.6
SBF043	Female	Semoq Bei	22	11.4	97%	12.5	4.69	26.6	84.8
SBF044	Female	Semoq Bei	39	9.5	81%	12.5	3.9	31.9	98.5
SBM023	Male	Semoq Bei	21	8.6	73%	17.5	5.46	32	99.4
SBM024	Male	Semoq Bei	39	9.0	76%	14.8	4.41	33.5	102.3
SBF045	Female	Semoq Bei	24	11.0	93%	12.8	4.27	30.1	92.1
SBM025	Male	Semoq Bei	25	9.6	81%	14.4	4.9	29.4	89.9
SBM026	Male	Semoq Bei	58	9.5	80%	15.1	4.76	31.7	97.6
SBM027	Male	Semoq Bei	51	8.8	75%	15.3	4.76	32.2	101.5
SBM028	Male	Semoq Bei	29	8.8	74%	16.1	5.36	30.1	94.3
SBM029	Male	Semoq Bei	18	11.6	98%	13	6.25	20.8	71
SBF046	Female	Semoq Bei	48	10.3	88%	13.8	5.34	25.8	84.5
SBM030	Male	Semoq Bei	35	9.3	79%	14.1	4.64	30.3	96.2
SBF047	Female	Semoq Bei	48	9.5	81%	14.3	5.06	28.2	88.5
SBF050	Female	Semoq Bei	51	8.0	68%	13.6	4.65	29.3	91.9
SBF051	Female	Semoq Bei	25	9.7	82%	12.3	4.85	25.3	81.2
SBF052	Female	Semoq Bei	20	13.2	112%	12.4	5.19	23.9	78.4
SBM053	Male	Semoq Bei	43	9.5	80%	15.4	4.71	32.8	101.2
STM001	Male	Temiar	29	10.1	86%	15.2	6.95	21.9	69.7
STM002	Male	Temiar	16	14.3	121%	12.3	5.2	23.6	75.1
STF098	Female	Temiar	37	13.6	116%	13.9	5.03	27.6	86.8
STF099	Female	Temiar	18	17.9	151%	10.9	5.86	18.6	61.5
STM003	Male	Temiar	53	15.7	133%	16.2	5.68	28.5	90
STM004	Male	Temiar	25	12.8	109%	14.6	5.47	26.7	83.3
STF100	Female	Temiar	43	11.0	93%	12.6	5.48	22.9	76.1

STM006	Male	Temiar	27	13.4	114%	14	5.55	25.2	81.6
STF101	Female	Temiar	40	16.3	138%	11.9	5.08	23.4	74
STF106	Female	Temiar	11	16.1	137%	13.4	5.19	25.9	80.9
STF107	Female	Temiar	6	16.0	136%	11.1	4.93	22.6	73.1
STM008	Male	Temiar	8	1.7	14%	11.9	4.28	27.8	85.7
STM009	Male	Temiar	10	14.2	120%	13.1	4.89	26.9	83.1
STF114	Female	Temiar	10	15.4	130%	12.1	4.72	25.5	78.8
STM011	Male	Temiar	22	11.3	95%	14.2	5.69	24.9	80.4
STM012	Male	Temiar	31	15.6	132%	15.1	5.71	26.4	81
STF116	Female	Temiar	39	10.0	85%	13.6	5.11	26.6	84.5
STM018	Male	Temiar	39	8.4	71%	15.5	4.82	32.2	98.2
STM019	Male	Temiar	40	8.6	73%	15.7	5.49	28.6	91.7
STM020	Male	Temiar	22	12.3	104%	14.8	5.76	25.8	79.4
STF120	Female	Temiar	11	1.3	11%	13.5	5	27	83
STF127	Female	Temiar	8	9.9	84%	11.8	4.38	27	80.5
STM024	Male	Temiar	29	10.0	85%	15.8	5.16	30.7	96.6
STM023	Male	Temiar	31	13.9	118%	14.4	5.39	26.7	85.2
STF130	Female	Temiar	36	18.0	152%	8.6	4.71	18.4	62.9
STF131	Female	Temiar	35	21.4	181%	7.8	5.42	14.4	51.6
STF132	Female	Temiar	25	13.6	115%	11.4	5.8	19.6	65.1
STF133	Female	Temiar	10	11.8	100%	12.4	4.59	27	85.3
STF139	Female	Temiar	28	15.5	132%	11.3	5.61	20.1	67.2
STM028	Male	Temiar	22	14.9	127%	14.8	5.53	26.6	82.8
STM029	Male	Temiar	74	14.9	127%	14.6	5.8	25.2	79.1
STM030	Male	Temiar	50	15.7	133%	13.5	5.22	25.8	81.2
STF140	Female	Temiar	46	15.7	133%	10.5	4.43	23.6	72.8
STF141	Female	Temiar	10	15.0	127%	12.4	5.11	24.2	74.1
STF142	Female	Temiar	42	10.3	87%	14.4	4.44	32.3	93.6
STM031	Male	Temiar	11	11.8	100%	11.3	4.71	24	76.3
STF143	Female	Temiar	24	13.0	110%	13	4.24	30.7	91.9
STF144	Female	Temiar	64	16.4	139%	14.1	5.38	26.3	81.5
STM032	Male	Temiar	30	13.9	118%	14.1	5.74	24.7	78
STM033	Male	Temiar	30	11.0	94%	16.4	6.44	25.5	78.6
STM034	Male	Temiar	26	14.8	125%	14.1	5.42	26	80.7
STM035	Male	Temiar	33	14.7	125%	14.8	5.61	26.3	84.1
STF145	Female	Temiar	26	10.1	86%	13.4	4.44	30.3	95.7
STM036	Male	Temiar	40	12.6	106%	13.4	6.29	21.3	70.5
STM037	Male	Temiar	43	2.1	17%	14	4.77	29.4	94
STM038	Male	Temiar	12	4.2	36%	10.5	5.31	19.8	63.2
STM039	Male	Temiar	6	4.0	34%	11.3	4.58	24.6	77.2
STF149	Female	Temiar	35	15.6	133%	8.3	4.8	17.3	60.5
STM044	Male	Temiar	39	9.7	82%	16.1	6.14	26.2	82.8
STF151	Female	Temiar	34	18.5	157%	7	4.26	16.4	59.7
STF153	Female	Temiar	42	6.4	54%	11.5	5.89	19.5	70.8
STF158	Female	Temiar	8	16.1	136%	11.8	5.03	23.5	73.8
STM049	Male	Temiar	61	14.9	126%	13.1	4.87	27	84.3
STF159	Female	Temiar	61	3.9	33%	12.1	3.94	30.8	95.9

STF160	Female	Temiar	45	7.4	63%	13.1	5.14	25.5	82.1
STM050	Male	Temiar	72	12.3	105%	11.5	4.93	23.3	76.1
STF161	Female	Temiar	20	4.1	35%	11.3	4.44	25.4	80.6
STM051	Male	Temiar	28	22.0	186%	12.1	6.32	19.2	62.1
STF163	Female	Temiar	17	19.3	164%	11.4	5.55	20.6	65.3
STM052	Male	Temiar	30	10.6	90%	14	4.6	30.3	92.1
STF165	Female	Temiar	28	15.6	132%	13.8	5.66	24.4	76.9
STM056	Male	Temiar	22	15.2	129%	15.7	6.53	24	75.4
STM062	Male	Temiar	12	16.8	143%	12.2	4.47	27.2	83.9
STM065	Male	Temiar	8	15.2	129%	10.8	4.52	23.8	76.3
STF167	Female	Temiar	59	12.6	107%	14.5	5.75	25.3	81.8
STF168	Female	Temiar	57	11.6	98%	14	4.33	32.3	100.9
STF174	Female	Temiar	8	16.2	137%	11.9	4.53	26.2	80.7
STF175	Female	Temiar	12	12.0	102%	12.5	4.41	28.3	89.9
STM068	Male	Temiar	5	16.0	135%	11.8	5.05	23.4	72.5
STF176	Female	Temiar	29	14.8	125%	13.8	5.38	25.5	79.4
STF178	Female	Temiar	33	9.3	78%	12.8	5.28	24.1	75.8
STM069	Male	Temiar	42	13.2	112%	13.2	5.48	24.1	76.5
STM070	Male	Temiar	24	9.9	84%	13.5	5.12	26.4	84.2
STM071	Male	Temiar	22	14.7	125%	15.4	5.92	26	80.8
STM072	Male	Temiar	41	13.1	111%	15.9	5.57	28.5	86.7
STF179	Female	Temiar	40	16.4	139%	13.8	5.65	24.5	76.7
STF180	Female	Temiar	45	13.4	113%	12.7	5.14	24.7	77.1
STM078	Male	Temiar	18	15.2	129%	17.7	6.83	25.9	80.2
STF097	Female	Temiar	19	11.9	101%	14.1	4.97	28.3	86.3
STM079	Male	Temiar	22	11.9	101%	15.1	5.01	30	92.6
STM080	Male	Temiar	37	14.0	119%	15.9	6.12	26	80.8
STF183	Female	Temiar	34	17.7	150%	10.8	4.76	22.7	73.7
STF186	Female	Temiar	24	14.0	119%	13	5.04	25.8	83
STF187	Female	Temiar	11	10.1	86%	12.9	4.73	27.3	88.1
STM085	Male	Temiar	51	15.2	128%	15	5.56	27	88.5
STF188	Female	Temiar	41	16.1	137%	12.6	5.75	21.9	69.9
STM089	Male	Temiar	54	16.7	141%	12.9	4.46	29	87.2
STM090	Male	Temiar	37	20.6	174%	13.2	6.67	19.8	63.3
STM091	Male	Temiar	19	13.7	116%	14.6	5.27	27.8	85.1
STF189	Female	Temiar	42	16.6	141%	11.8	4.82	24.5	75.8
STF190	Female	Temiar	37	18.3	155%	10.2	5.58	18.2	60.9
STF191	Female	Temiar	24	19.0	161%	11.9	5.71	20.9	67.4
STF192	Female	Temiar	22	15.9	135%	11.6	5.03	23.1	73.4
STM096	Male	Temiar	9	14.3	121%	13	4.35	29.9	90.2
STF201	Female	Temiar	31	17.0	144%	13.7	6.32	21.7	70.8
