# Supplementary material for: Prevalence and molecular heterogeneity of glucose-6-phosphate dehydrogenase (G6PD) deficiency in the Senoi Malaysian Orang Asli population
Source: PLoS One. 2023 Dec 12;18(12):e0294891. doi: 10.1371/journal.pone.0294891 (PMC10715666; doi:10.1371/journal.pone.0294891)
Supplement: Attachment — Submitted filename: Review Pone D23-01672.docx [file pone.0294891.s002.docx]

**Review of the manuscript: “Prevalence and molecular heterogeneity of glucose-6-phosphate dehydrogenase (G6PD) deficiency in the Senoi Malaysian Orang Asli population” (PONE-D-23-01672)**

Summary: This article reports the results of a survey among one of Malaysia’s ethnicity (Senoi Malaysian Orang Asli) on the prevalence and dominant genotypes of G6PD deficiency. A total of 662 participants were screened by phenotypic test and all those identified as deficient (n=87, 13%) were genotyped. Among all phenotypic deficient participants, seven G6PD variants were identified. The authors conclude that the prevalence of G6PD deficiency was very high among the study population.

General: The addressed question is relevant for the local context. My key concerns are:

- The authors report overall prevalence of G6PD deficiency within the target population but do not provide any details on how participants were recruited. If participants were not recruited at random and multiple members of the same family were enrolled, the reported prevalence will be biased. The authors need to report prevalence together with its variation (95% confidence interval or interquartile range)
- The authors do not explain their statistical analysis in the methods section, some information is provided in the results section that needs to be moved accordingly. From the results section I understand that the authors calculated the mean G6PD activity after excluding everyone with activities below the manufacturers recommended range of normal activities (<7.8U/gHb). This mean was then defined as 100% activity and anyone with <10% activity was categorised as severe and anyone with >10% activity and <60% was defined as moderate deficient. I recommend that the authors calculate the adjusted male median (AMM) [1] instead and define anyone with activities below 30% of the AMM as deficient and anyone with activities >30% and <70% of the AMM as having intermediate activities. This is the approach chosen by the majority of comparable articles (for example [2]). I am also unsure why the authors stratify their analysis by age group? I am not aware that G6PD activity would differ between 2–12-year-old and older individuals? The here observed difference is likely due to chance.
- Genotyping only phenotypically deficient individuals introduces a bias. Is there any chance the authors can also genotype a random subset of phenotypically G6PD normal individuals?

Minor comments:

- Can the authors add definitions of G6PD deficiency in the abstract?
- Can the authors add total numbers of males and females enrolled in the abstract to put the proportion of deficinet males and females into perspective?
- In the abstract pervalence of G6PD deficinecy is mentioned twice, in lines 32 and 34, kindly streamline
- In the abstract, can the authors start of by stating that no mutation was found among 18 genotyped and deficnet indidvausl (if I counted correct).
- In the abstract can the authors include how many individuals were found to be hemi/homo or heterozygous?
- Line 38 and following, can the authors add absolute numbers to the proportions?
- Line 48: this statement requires a reference.
- Line 52: replace “disease” by “condition”?
- Line 53: G6PD deficient indivduals can be found in almost all populations, not only the mentioned continents.
- Line 74 states that Primaquine is the most widely used antimalarial and this statement will need a reference.
- Line 77 states: “Despite the high susceptibility and endemicity of malaria among the Orang Asli in Malaysia” – can the authors clarify what susceptoibility refer to?
- Table 3: can the authors add that this table only covers G6PD normals? Why not include deficients as a comparisson as well?
- Line 173: how was the level of significance calculated? Please add this to the methods.
- Table 5: rather than categorising all variants as “moderate” or “severe”, present range of observed activities / variant
- Table 5: In the “total: column there is a mention of “21+2” etc. and it is unclear what this means. Is there a footnote missing?
- Throughout the text the authors refer to a variation as ± (for example 14.33±3.48 in line 36) and this is ambiguous. Can the authors replace this by mean and 95%CI for normal distributed data and median and IQR for not normally distributed data?
- Were all partricipanst afebrile and free of malaria? If yes, kindly add.
- Throughout the entire article, the authors should include manufacturer and country of the manufacturer whenever mentioning a product.

Language: the language of the manuscript must be revised by a native English speaker familiar with the topic

Reference:

1.	Domingo, G.J., et al., *G6PD testing in support of treatment and elimination of malaria: recommendations for evaluation of G6PD tests.* Malar J, 2013. **12**: p. 391.

2.	Ley, B., et al., *Wide range of G6PD activities found among ethnic groups of the Chittagong Hill Tracts, Bangladesh.* PLOS Neglected Tropical Diseases, 2020. **14**(9): p. 8697--1.
