# Supplementary material for: Prevalence and molecular heterogeneity of glucose-6-phosphate dehydrogenase (G6PD) deficiency in the Senoi Malaysian Orang Asli population
Source: PLoS One. 2023 Dec 12;18(12):e0294891. doi: 10.1371/journal.pone.0294891 (PMC10715666; doi:10.1371/journal.pone.0294891)
Supplement: Attachment — Submitted filename: Response to Reviewers.docx [file pone.0294891.s003.docx]

**Authors’ Response**

**Title of the manuscript:**

**“Prevalence and molecular heterogeneity of glucose-6-phosphate dehydrogenase (G6PD) deficiency in the Senoi Malaysian Orang Asli population” (PONE-D-23-01672)**

| **Editor** | |
| --- | --- |
| **Comments** | **Authors’ Response** |
| 1. Please ensure that your manuscript meets PLOS ONE's style requirements, including those for file naming.  2. Please remove any funding-related text from the manuscript and let us know how you would like to update your Funding Statement.  3. All PLOS journals require that the minimal data set be made fully available. Upon re-submitting your revised manuscript, please upload your study’s minimal underlying data set as either Supporting Information files or to a stable, public repository and include the relevant URLs, DOIs, or accession numbers within your revised cover letter. We will update your Data Availability statement to reflect the information you provide in your cover letter. | 1. Thank you for the comments. We have checked though the PLOS ONE's style requirements and made necessary amendments, including the file names. 2. We apologize for the tehcnical issue. We have now removed the funding-related text from the manuscript (the Acknowledgement section). Thank you, we agreed with the current Funding Statement and no further updates are required from our side. 3. We have uploaded our study’s minimal underlying data set as Supporting Information file 1 (S1_file.pdf). We have fully anonymized the data and removed any potentially identifying patient information. Thank you. |

| **Reviewer 1** | |
| --- | --- |
| **Comments** | **Authors’ Response** |
| **GENERAL**  1. The authors report overall prevalence of G6PD deficiency within the target population but do not provide any details on how participants were recruited. If participants were not recruited at random and multiple members of the same family were enrolled, the reported prevalence will be biased. The authors need to report prevalence together with its variation (95% confidence interval or interquartile range) | Thank you for your valuable comments. The authors have exluded the family members in calculation of the overall prevalence to prevent bias. We have recalculated and provided a revised prevalence, as well as its variation (95% confidence interval). These results were written in abstract as well as in the body of the manuscript.  In the abstract:  **“…The overall prevalence of G6PD deficiency was 15.2% (95% Confidence Interval: 11-19%; 56 of 369) , with males (30 of 172; 17.4%) outnumbering females (26 of 197; 13.2%). The adjusted male median (AMM), defined as 100% G6PD activity, was 11.8 IU/gHb. A total of 36 participants (9.6%; 26 male and 10 female) were severely deficient (<30% of AMM) and 20 participants (5.4%; 4 male and 16 female) were G6PD-intermediate (30 - 80% of AMM).”** |
| 2. The authors do not explain their statistical analysis in the methods section, some information is provided in the results section that needs to be moved accordingly. From the results section I understand that the authors calculated the mean G6PD activity after excluding everyone with activities below the manufacturers recommended range of normal activities (<7.8U/gHb). This mean was then defined as 100% activity and anyone with <10% activity was categorised as severe and anyone with >10% activity and <60% was defined as moderate deficient. I recommend that the authors calculate the adjusted male median (AMM) instead and define anyone with activities below 30% of the AMM as deficient and anyone with activities >30% and <70% of the AMM as having intermediate activities. This is the approach chosen by the majority of comparable articles (for example [2]). I am also unsure why the authors stratify their analysis by age group? I am not aware that G6PD activity would differ between 2–12-year-old and older individuals? The here observed difference is likely due to chance. | Thank you for this comment. We have added the explaination on statistical analysis in the methods section (Data analysis) as suggested.  We appreciate the Reviewer’s recommendation. We have amended our calculation using the adjusted male median (AMM). We have also used the cut-off threshold of 30% and 70% as per advised.  We agreed with the comments given regarding the age. We have amended our manuscript and did not stratify the analysis by age group. |
| 3. Genotyping only phenotypically deficient individuals introduces a bias. Is there any chance the authors can also genotype a random subset of phenotypically G6PD normal individuals? | Thank you for this comment. We have genotyped a random subset of phenotypically G6PD normal individuals. We have now included this point in our manuscript.  **“Molecular analysis was performed on 87 samples including all G6PD-deficient blood samples (n=56) and subset of G6PD-normal sample (n=31).”** |
| **MINOR**   1. Can the authors add definitions of G6PD deficiency in the abstract? 2. Can the authors add total numbers of males and females enrolled in the abstract to put the proportion of deficinet males and females into perspective? 3. In the abstract pervalence of G6PD deficinecy is mentioned twice, in lines 32 and 34, kindly streamline 4. In the abstract, can the authors start of by stating that no mutation was found among 18 genotyped and deficnet indidvausl (if I counted correct). 5. In the abstract can the authors include how many individuals were found to be hemi/homo or heterozygous? 6. Line 38 and following, can the authors add absolute numbers to the proportions? 7. Line 48: this statement requires a reference. 8. Line 52: replace “disease” by “condition”? 9. Line 53: G6PD deficient indivduals can be found in almost all populations, not only the mentioned continents. 10. Line 74 states that Primaquine is the most widely used antimalarial and this statement will need a reference. 11. Line 77 states: “Despite the high susceptibility and endemicity of malaria among the Orang Asli in Malaysia” – can the authors clarify what susceptoibility refer to? 12. Table 3: can the authors add that this table only covers G6PD normals? Why not include deficients as a comparisson as well? 13. Line 173: how was the level of significance calculated? Please add this to the methods. 14. Table 5: rather than categorising all variants as “moderate” or “severe”, present range of observed activities / variant 15. Table 5: In the “total: column there is a mention of “21+2” etc. and it is unclear what this means. Is there a footnote missing? 16. Throughout the text the authors refer to a variation as ± (for example 14.33±3.48 in line 36) and this is ambiguous. Can the authors replace this by mean and 95%CI for normal distributed data and median and IQR for not normally distributed data? 17. Were all partricipanst afebrile and free of malaria? If yes, kindly add. 18. Throughout the entire article, the authors should include manufacturer and country of the manufacturer whenever mentioning a product. | Thank you for the excellent comments. We have amended our manuscript accordingly:   1. We have added definitions of G6PD deficiency in the abstract.   **“Glucose-6-phosphate dehydrogenase (G6PD) deficiency is an X-linked genetic disorder characterized by reduced G6PD enzyme level in the blood.”**   2. We have added the total numbers of males and females enrolled in the abstract.   **“…A total of 662 blood samples (369 males and 293 females).”**   3. We have amended the lines mentioned and streamlined the details. 4. Excellent suggestion. We have included this point as suggested. Yes, the number given (n=18) was correct.   **“A total of 87 samples were genotyped, of which 18 showed no mutation.”**   5. We have included the number of participants with the hemi/homo or heterozygous in the abstract as suggested.   **“Our analysis revealed 27 hemizygote males, 18 heterozygote females, 7 homozygote females, and 2 compound heterozygote females.”**   6. In lines 38 (abstract), the authors have added absolute numbers to the proportions as requested.   **“…Seven mutations were found among 69 genotyped samples; IVS11 T93C (47.1%; n=41), rs1050757 (3’UTR +357A>G)(39.1%; n=34), G6PD Viangchan (c.871G>A)(25.3%; n=22), G6PD Union (c.1360C>T)(21.8%; n=19), c.1311C>T(20.7%; n=18), G6PD Kaiping (c.1388G>A)(8.0%; n=7), and G6PD Coimbra (c.592C>T)(2.3%; n=2).**   7. The authors have added a suitable reference for this statement.   **Luzzatto L, Ally M, Notaro R. Glucose-6-phosphate dehydrogenase deficiency. Blood. 2020;136(11): 1225–1240. doi: 10.1182/blood.2019000944**   8. We have replaced the word “disease” with “condition”. 9. We agreed with this comment, and we have amended the sentence accordingly to address this point.   **“This X-linked hereditary condition affects the entire world, but it is more prevalent particularly in parts of the African continent, the Middle East, and Southeast Asia, where it often overlaps with the geographical distribution of malaria infection.”**   10. We have added a reference for this statement.   **Camarda G, Jirawatcharadech P, Priestley RS, Saif A, March S, Wong MH, Leung S, et al. Antimalarial activity of primaquine operates via a two-step biochemical relay. Nature communications. 2019 Jul 19;10(1):3226.**   11. We have added a sentence to further clarify the point of high susceptibility.   **“…The Orang Asli population is highly susceptible to mosquito bites and malaria infection due to their isolated settlements in tropical forests and traditions of hunting and foraging for food in the jungle….”**   12. Thank you for this excellent comment. We have amended the table accordingly (Refer Tables 3 and 4). 13. We have added the method for calculation of significance in the “Data Analysis” section. 14. We have amended the table and presented the range of observed activities as suggested (Refer Table 5). 15. We have added the footnote missing from the table.   **“Footnote: 1^a^ refers to a compound heterozygote case of G6PD Viangchan + Union, and 1^b^ refers to a compound heterozygote case of G6PD Kaiping + Union”**   16. Thank you for the comment. We have included the 95%CI for our AMM value and G6PD levels for all genotypes in Table 5. However, for other values throughout the text, we would like to respectfully propose using the mean and standard deviation (SD) (example: 14.33±3.48 ) as measures of variation, rather than the 95% confidence interval (CI), to represent our data. We believe that the mean and SD provide a clear and concise way to describe the central tendency and spread of our data, without the additional complexity introduced by the CI. The use of these statistics aligns with common reporting practices in our field and maintains the readability of our manuscript. 17. Yes, all participants were afebrile and free of malaria. We have added this information in the “Result” section.   **“The participants were healthy, afebrile, and free of malaria.”**   18. Thank you for this insightful comment. We have checked the entire manuscript and included the details of the manufacturer and country of the manufacturer for all products mentioned. All details were provided in the first mention, and these details were not included in subsequent mention throughout the manuscript (aligned with the journal’s guideline). |
| Language:  The language of the manuscript must be revised by a native English speaker familiar with the topic  Reference:  1. Domingo, G.J., et al., G6PD testing in support of treatment and elimination of malaria: recommendations for evaluation of G6PD tests. Malar J, 2013. 12: p. 391.  2. Ley, B., et al., Wide range of G6PD activities found among ethnic groups of the Chittagong Hill Tracts, Bangladesh. PLOS Neglected Tropical Diseases, 2020. 14(9): p. 8697--1. | 1. Thank you for this suggestion for language improvement. We have conducted a thorough grammar check and revision of the manuscript to ensure its clarity and readability. 2. Thank you for this useful comment. We have included the relevant references in our reference list. |

| **Reviewer 2** | |
| --- | --- |
| **Comments** | **Authors’ Response** |
| 1. This is a well written paper with clear and concise findings. I only have minor edits to suggest before moving this forward to publication.  Ln 24 italicize plasmodium  Ln 31 subethnic group  Ln 76 P. falciparum | Thank you for reviewing our manuscript and giving valuable comments. We have amended all mentioned points accordingly. |
| 2. Were the quantitative assays run in duplicate? I am unfamiliar with this kit, it might be valuable to include any literature comparing this kit with Point scientific or trinity biotech G6PD spectrophotometric tests.  General comment – be consist with use of sub-ethnic vs subethnic - same goes for subethnicity | Thank you for this comment. The quantitative assays were not run in duplicate for our study. While trying our best, we have included a statement in addresing this point with inclusion of several relevant references.  “**In numerous earlier research, the OSMMR-2000D kit assay served as the gold standard quantitative G-6-PD enzymatic test. [18,19,25 – 28].”**  Thank you for this comment. We have checked through the manuscript and be consistent in using subethnic and subethnicity (13 replacements). |
| 3. How did normal G6PD thresholds match up with the study by Pfeffer et al. “Quantification of glucose-6-phosphate dehydrogenase activity by spectrophotometry: A systematic review and meta-analysis” I’d be interested in seeing if they aligned, given the unique population of this stud | Thank you for your high interest. We appreciate your comment.  In our study, the normal threshold for 100% G6PD activity corresponded to a slightly higher value of 11.8 IU/gHb. The mentioned paper by Pfeffer et al. (2020) stated that a universal threshold of 100% G6PD activity was defined as 9.4 IU/gHb. However, the authors also highlighted that caution is stricly advised in comparing findings based on absolute G6PD activity measurements across studies due to variability in laboratory methods, with possible contribution of unmeasured population factors.  We have briefly included this interesting point and the mentioned reference in our manuscript as well.  **“The normal threshold for 100% G6PD activity, the AMM, of our study corresponded to a slightly higher value of 11.8 IU/gHb compared to a universal threshold of 9.4 IU/gHb, as described by a meta analysis study of Pfeffer et al. (2020) [25]. However, as described by the article, researchers should strongly cautioned against drawing conclusions solely on absolute G6PD activity from different studies, due to interlaboratory variability and possible unmeasured population factors.”**  Thank you. |
